# Protective Effect of Resveratrol against Hypoxia-Induced Neural Oxidative Stress

**DOI:** 10.3390/jpm12081202

**Published:** 2022-07-23

**Authors:** Amogh Auti, Nicola Alessio, Andrea Ballini, Mario Dioguardi, Stefania Cantore, Salvatore Scacco, Antonio Vitiello, Lucio Quagliuolo, Barbara Rinaldi, Luigi Santacroce, Marina Di Domenico, Mariarosaria Boccellino

**Affiliations:** 1 Department of Precision Medicine, University of Campania “Luigi Vanvitelli”, 80138 Naples, Italy; amogh.auti@unicampania.it (A.A.); lucio.quagliuolo@unicampania.it (L.Q.); marina.didomenico@unicampania.it (M.D.D.); mariarosaria.boccellino@unicampania.it (M.B.); 2 Department of Experimental Medicine, University of Campania “Luigi Vanvitelli”, 80138 Naples, Italy; nicola.alessio@yahoo.it (N.A.); barbara.rinaldi@unicampania.it (B.R.); 3 Department of Clinical and Experimental Medicine, University of Foggia, 71122 Foggia, Italy; mario.dioguardi@unifg.it; 4 Independent Researcher, 70129 Bari, Italy; 5 Department of Basic Medical Sciences, Neurosciences and Sense Organs, University of Bari ALDO MORO, 70124 Bari, Italy; salvatore.scacco@uniba.it; 6 Pharmaceutical Department, Local Sanitary Unit Umbria 1, 06132 Perugia, Italy; antonio.vitiello2@uslumbria1.it; 7 Microbiology and Virology Unit, Department of Interdisciplinary Medicine, School of Medicine, University of Bari ALDO MORO, 70124 Bari, Italy; luigi.santacroce@uniba.it; 8 Department of Biology, College of Science and Technology, Temple University, Philadelphia, PA 19122-6078, USA

**Keywords:** PC12 cells, resveratrol, oxidative stress, ischemia, hypoxia, personalized medicine, translational research

## Abstract

Oxidative stress plays an important role in brain aging and in neurodegenerative diseases. New therapeutic agents are necessary to cross the blood–brain barrier and target disease pathogenesis without causing disagreeable side effects. Resveratrol (RSV) may act as a neuroprotective compound, but little is known about its potential in improving the cognitive and metabolic aspects that are associated with neurodegenerative diseases. The objective of this study was to investigate the protective effects and the underlying mechanisms of RSV against hypoxia-induced oxidative stress in neuronal PC12 cells. For the induction of the hypoxia model, the cells were exposed to oxygen-deprived gas in a hypoxic chamber. Cell cycle and apoptosis were analyzed by a fluorescence activated cell sorting (FACS) analysis. The intracellular reactive oxygen species (ROS) level was analyzed by using dichlorodihydrofluorescein diacetate (DCFDA) and 5-(and-6)-chloromethyl-2’,7’-dichlorodihydrofluorescein diacetate, acetyl ester (CM-H2DCFDA) tests. The expression of activated caspase-3, -9, Bcl-2, Bax, p53, and SOD was investigated by a Western blot analysis. We found that hypoxia reduced PC12 viability by inducing apoptosis, while RSV treatment attenuated the ROS-induced damage by reducing caspase-3, -9, and the Bax/Bcl-2 ratio. The RSV treated groups were found to improve cellular health, with a 7.41% increase in the S phase population in the 10 µM group, compared to the control. Hence, RSV has a protective effect in neuronal cells and may halt the cell cycle in the G1/S phase to repair the intracellular damage. Therefore, RSV could be a good candidate to act as an antioxidant and promising preventive therapeutic agent in neurodegenerative diseases for personalized medicine.

## 1. Introduction

Reactive oxygen species (ROS) are produced continuously in organisms during normal cellular activity. In all cells, in a state of normal physiology, there is a balance between antioxidants and pro-oxidants. Therefore, if the level of antioxidants is reduced or the level of pro-oxidants increases, serious damage to the cell ensues. This imbalance and the inability of cells to protect against them is called oxidative stress (OS) [1]. OS plays an important role in brain aging, neurodegenerative diseases, and other related adverse conditions, such as ischemia [2,3,4,5]. The central nervous system (CNS), compared to other tissues, is particularly vulnerable to OS, because of its higher oxygen consumption, high lipid content and insufficient antioxidant defense systems. Oxidative stress in the CNS causes damage to DNA, proteins and lipid peroxidation. In addition, it induces necrosis and apoptosis, which are two main causes of neuronal death [6]. The excessive production of ROS and insufficient activity of antioxidant defense mechanisms have been implicated in the pathogenesis of many neurodegenerative diseases, including Parkinson’s disease (PD), Multiple Sclerosis (MS), Alzheimer’s disease (AD), Huntington disease (HD), and amyotrophic lateral sclerosis (ALS) [7].

Oxygen and glucose are the principal metabolites that support a normal neuronal metabolism. Prolonged hypoxia results in the irreversible loss of neurons, due to necrosis and apoptosis causing neurodegenerative pathologies such as Alzheimer’s disease (AD) [8]. Lack of oxygen limits energy metabolism to glycolysis and lowers the overall adenosine triphosphate (ATP) demand [9,10]. The axonic neurons try to adapt to the brief hypoxia through ATP demand, which falls by 40%, but prolonged hypoxia leads to cell death, due to a lack of adequate ATP level [11]. The switch from oxygen-dependent oxidative phosphorylation processes to anaerobic metabolism potentially triggers a significant energy imbalance and the production of ROS. The concentrated metabolic flux in glycolysis results in increased ROS levels [12,13]. In the enzymatic antioxidant defense, the essential components are superoxide dismutase (SOD), catalase (CAT), glutathione peroxidase (GPx), and glutathione reductase (GR), while the non-enzymatic antioxidants include glutathione (GSH), thioredoxin (Trx), vitamins A, E, and C, and phenolic compounds.

The increase in average life expectancy has led to the growth of neurodegenerative diseases and the existing treatments are only symptomatic. New therapeutic agents are necessary to cross the blood–brain barrier and target disease pathogenesis without causing disagreeable side effects. Thus, early intervention—preferably pre-clinically when the brain is not significantly affected—is a better option for effective treatment.

Natural products have a wide range of pharmacological or biological activity, making them proper candidates in translational research, as well for the treatment of neurological disorders and neurodegenerative diseases [14,15,16,17,18]. Several OMICS studies on the discovery and identification of new neuroprotective drugs have shown that natural bioactive compounds are possible neuroprotective agents against various types of neurodegenerative disorders [19]. Resveratrol (3,4,5-trihydroxystilbene, RSV) is a small polyphenol in a white powder form at a melting point of 253–255 °C with a low weight of about 228.24 Da [20]. Sources of RSV include various berries, red grapes and grape wines in different concentrations [21,22,23,24]. Thanks to its pleiotropic protective action against OS, inflammation and cancer, RSV has gained considerable interest as a possible treatment for chronic human diseases [25]. An in vivo study on RSV with a flavonoid showed that it crossed the blood–brain barrier and conferred neuroprotective effects. Hence, it can be used to treat neuronal disorders [26]. Moreover, additional experimental evidence has shown that RSV can preserve cognitive functions and, thus, offer protection against neurodegeneration [27]. Cells that are treated with RSV alter the intracellular transcription levels of cyclins and cyclin-dependent kinases (which modulate the cell cycle), as well as control apoptosis by modulating FAS/FAS-Ligand, caspase-3 and -9 activity [28]. Despite resveratrol’s increasing popularity, its molecular mechanisms of action are still poorly understood.

The objective of this translational study was to investigate the protective effects and the underlying mechanisms of RSV against hypoxia-induced oxidative stress in neuronal PC12 cells.

## 2. Materials and Methods

### 2.1. Cell Culture, Resveratrol Treatment, and Hypoxia

The PC12 cells were cultured in RPMI 1640 medium with glutamine (Gibco; Thermo Fisher Scientific, Inc., Waltham, MA, USA) with 10% fetal bovine serum (Gibco, Thermo Fisher Scientific, Inc.) and 15% donor horse serum (Gibco, Thermo Fisher Scientific, Inc.), and then cultured at 37 °C in an atmosphere containing 5% CO_2_ in a humidified incubator.

The PC12 cells were treated with two concentrations of RSV (Sigma Aldrich, R5010, St. Louis, MO, USA) (2.5 and 10 µM in 0.05% DMSO in RPMI 1640 medium), and with an appropriate control for 24 h at 37 °C before hypoxic induction. For induction of the hypoxia model, after culture at 37 °C with 5% CO_2_ for 24 h, the PC12 cells were placed in a hypoxic chamber (Billups-Rothenburg, Del Mar, CA, USA) and exposed to oxygen-deprived gas (1% O_2_, 5% CO_2_, 92% N_2_) for 6 h, as reported [29,30]. The cells were then cultured under normal conditions for 24 h and harvested for further analysis.

### 2.2. Cytotoxicity Assay

The proliferation assay was performed in normoxic (standard culture conditions) to assess the adverse effect of RSV on the PC12 cells. Firstly, the PC12 cells were counted, and approximately 1 × 10^4^ cells per well were seeded in a 96-well cell culture plate (Corning Inc., Corning, NY, USA). After incubation at 37 °C in a humidified atmosphere with 5% CO_2_ for 24 h, the culture medium was replaced with fresh medium, and a series of concentrations of RSV (20–100 µM) that were diluted with 0.05% DMSO in RPMI 1640 were added. Four replicates were made for each concentration. A total of 10 μL of the CCK-8 reagent (MedChemExpress Ltd., New York, NY, USA) was added into each well with fresh media, and OD at 450 nm was measured using a multifunction microplate reader (BioRad, Hercules, CA, USA) at 24, 48 and 72 h of incubation.

### 2.3. Annexin V Assay

Apoptotic cells were detected using a fluorescein that was conjugated with an Annexin V kit on a Guava easyCyte (Millipore, Milano, Italy) flow cytometer, following the manufacturer’s instructions, as described [31,32]. The kit used two separate dyes (Annexin V and 7AAD) to identify apoptotic and non-apoptotic cells [33]. Annexin V (red) binds to phosphatidylserine on the external membrane of apoptotic cells, while 7AAD (blue) permeates and stains the DNA of late-stage apoptotic and dead cells. Staining allows the identification of three cell populations: non-apoptotic cells (Annexin V− and 7AAD−); early apoptotic cells (Annexin V+ and 7AAD−); and late-apoptotic or dead cells (Annexin V+ and 7AAD+). In our experimental conditions, the early and late apoptotic cells were grouped together. We also analyzed cells with and without hypoxic treatment for the sake of comparison.

### 2.4. Reactive Oxygen Species Detection

The intracellular level of ROS is an important biomarker for oxidative stress, and an increased ROS level generally indicates a threat to genomic integrity. The production of ROS was estimated with the fluorescent dye 2′,7′-dichlorodihydrofluorescein diacetate (H2DCFDA). H2DCF is non-fluorescent, but in the presence of intracellular ROS it is oxidized to highly fluorescent dichlorofluorescein (DCF). The intracellular ROS level in the control and treated PC12 was analyzed by using the dichlorodihydrofluorescein diacetate (DCFDA) assay (Thermo Fisher Italia, Monza, Italy), according to the manufacturer’s instructions. The ROS derivatives were quantified on a Guava easyCyte flow cytometer (Merck Millipore, Burlington, MA, USA), following the manufacturer’s instructions. These stained cells were spread on a glass slide and stained with DAPI. Next, these slides were observed with a fluorescent microscope (Nikon Eclipse Ci) (Ex-465–495 nm) under low light conditions to reduce photo-bleaching. The image analysis and processing were carried out using NIS-Element analysis software (Nikon, Milan, Italia).

### 2.5. Cell Cycle Analysis

In total, 5 × 10^4^ PC12 cells were collected and fixed in 70% ethanol at −20 °C, overnight, followed by PBS 1X washes, and finally dissolved in a hypotonic buffer containing propidium iodide (PI) (Sigma-Aldrich, St. Louis, MO, USA). Samples were acquired on a Guava easyCyte flow cytometer (Merck Millipore, Burlington, MA, USA), and analyzed with a standard procedure using easyCyte software, as described [34].

### 2.6. Western Blotting

The cells were lysed in a buffer containing 0.1% Triton-X100 (Roche, Basel, Switzerland) for 30 min in ice, and 20 μg of each lysate was electrophoresed in a polyacrylamide gel and electroblotted onto a nitrocellulose membrane, as described [35]. All the primary antibodies were used according to the manufacturers’ instructions. Immunoreactive signals were detected with a horseradish peroxidase-conjugated secondary antibody (SantaCruz, CA, USA). Primary antibodies used: anti-tubulin, anti-Bcl-2, anti-Bax, anti-p53, anti-SOD, anti-caspase-3 (cleaved), and anti-caspase-9 (cleaved), which were obtained from Santa Cruz Biotechnology (SantaCruz, CA, USA). After washing with tris-buffered saline/0.10% Tween-20, the blots were incubated with Goat anti-Mouse IgG H&L (Santa Cruz Biotechnology (SantaCruz, CA, USA) for 2 h at room temperature. Tubulin was detected using mouse monoclonal anti-tubulin antibody, as elsewhere described [36] (Sigma-Aldrich). All the antibodies were used according to the manufacturer’s instructions. Protein blot signals were visualized with enhanced chemiluminescence (Western Bright ECL; Witec AG, Luzern, Switzerland), and imaged with a detection system (Chemidoc XRS+, Bio-Rad, Cressier, Switzerland). A densitometric analysis of chemiluminescent signals was performed with the QuantityOne (BioRad) software using the total protein, as described [37].

### 2.7. Statistical Analysis

The statistical analysis and graphs were performed using GraphPad Prism 7.00 software. All data were expressed as mean ± standard deviation (SD). The number of experiments that were performed are mentioned within the figure legends. The statistical significance of the differences between multiple groups was determined using a one-way ANOVA. The statistical significance was defined as *p* < 0.05 (* *p* < 0.05, ** *p* < 0.01). A post-hoc test (Tukey test) was used to interpret the ANOVA results.

## 3. Results

### 3.1. Hypoxia-Induced Cellular Toxicity

**Figure 1:**
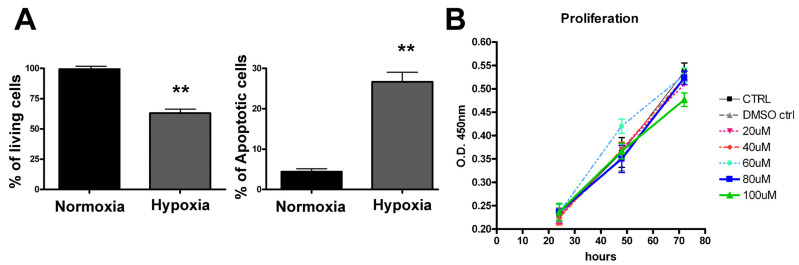
*Hypoxic treatment induces cell death in PC12 cells.* PC12 cells were seeded in two 6 well plates and subjected to hypoxia in an airtight chamber supplied with hypoxic gases for 6 h at 37 °C. The other plate was maintained at standard culture conditions (normoxia). Post-hypoxic treatment: both the culture plates were maintained in fresh medium at standard culture conditions and harvested after for 24 h. (**A**) Apoptotic cells were detected using a fluorescein conjugated with Annexin V kit. The bar graph represents quantification in percentage values of live cells and the apoptotic cell death comparison between normoxic vs. hypoxia-treated cells. (**B**) The cytotoxic effect of RSV was analyzed by CCK-8 assay. Data expressed as mean ± SD (** *p* < 0.01) of triplicate experiments.

First, we evaluated the effect of hypoxic stress, as well as RSV toxicity, on a PC12 neuronal cell model. The two groups, normoxic cells and hypoxia-treated cells, were analyzed for the primary effect of hypoxic condition. The cells were placed in the hypoxic chamber for 6 h and subsequently cultured under normal culture conditions for 24 h. Finally, the cells were trypsinized, washed with PBS and analyzed by annexin V-FITC/PI assay using a fluorescence activated cell sorting (FACS) analysis. As shown in Figure 1A, hypoxic stress induced approximately 30% (*p* < 0.01) cell death due to apoptosis, while cells that were grown under standard culture conditions showed an insignificant rate of apoptosis (less than 5%). To rule out the RSV toxicity, we examined a range of RSV concentrations (20, 40, 60, 80, 100 μM) on unstressed PC12 cells by using a CCK-8 cell proliferation assay. We observed that the highest concentration of RSV had no significant toxicity on PC12 cells over the 48 and 72 h of incubation (Figure 1B). Therefore, to assess the antioxidant effect of RSV on PC12 cells under hypoxia, we used the lowest concentrations of RSV (2.5 μM, 10 μM) in a further analysis.

### 3.2. RSV Attenuates Hypoxia-Induced Cell Apoptosis in PC12 Cells

**Figure 2:**
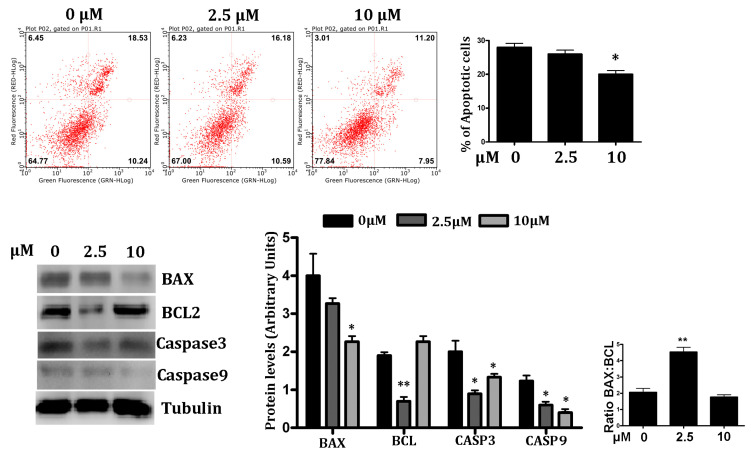
*Effect of RSV on hypoxia-induced apoptosis in PC12 cells*. **Upper Panel**—Each quadrant of FACS analysis shows proportion of cells (values in %). Lower left quadrant (absence of both markers) indicates viable cells; upper left quadrant (PI positive) indicates cellular necrosis; upper right quadrant (Annexin V positive and PI positive) indicates late-stage apoptosis; lower right quadrant (Annexin V positive) indicates early-stage apoptosis. The bar graph represents the mean % of late apoptotic cells in RSV treated under hypoxic conditions, (*n* = 3), (* *p* < 0.05, ** *p* < 0.01). **Lower Panel**—Western blot analysis of apoptotic regulator proteins. Quantification of Bcl-2, Bax, both cleaved caspase-3 and caspase 9 expression were presented in bar graphs as the fold-increase, respectively. All protein expression values were neutralized by tubulin expression, which was used as internal control. Ratio of Bax/Bcl-2 is represented in bar graph (Extreme right). Data are represented as means ± SD (*n* = 3), (* *p* < 0.05, ** *p* < 0.01) of triplicate experiments.

We explored the hypoxia-induced effect on PC12 cells in the presence of different concentrations of RSV (2.5 and 10 μM) to assess the recovery from programmed cell death. The cells were pre-treated with RSV (2.5 and 10 μM) for 24 h and then incubated in a hypoxic chamber for 6 h and assessed for apoptosis by an Annexin V-FITC/PI assay, followed by a flow cytometry analysis. As shown in Figure 2 (upper panel), the control showed significant damage in response to hypoxia (*p* < 0.05); the early and late apoptosis rates were about 28%. The treatment with RSV improves cell survival by increasing the number of live cells by 13% and lowering the number of dead cells by 10%. This phenomenon is particularly significant at 10 μM of RSV, compared to the control.

The activation of caspases is a critical event in the proteolytic cascade elicited by apoptotic stimuli. To further examine the molecular mechanisms of the protective effect of RSV in PC12 cells in hypoxic conditions, we investigated the level of activated caspase-3 and -9 by a Western blot analysis. Consistent with the reduction in apoptosis, we found that caspase-3 and caspase-9 were significantly down-regulated (*p* < 0.05) in RSV-treated PC12 cells, compared to the control (Figure 2, bottom panel).

Next, we examined whether the protective effect of RSV involved the modulation of Bcl-2 family proteins’ expression by a Western blot analysis. Proapoptotic protein Bcl-2-associated X protein (Bax) and antiapoptotic protein B-cell lymphoma-2 (Bcl-2) were evaluated. In particular, Bax and Bcl-2, two vital proteins of apoptosis, were blotted to assess the intracellular events. We found that the RSV treatment of PC12 cells in hypoxic conditions downregulated Bax expressions, and concomitantly upregulated antiapoptotic protein expression Bcl-2 (Figure 2, bottom panel). The Bax/Bcl2 ratio was lower in 10 µM of RSV (1.88) than the control cells (2.03).

### 3.3. Antioxidant Effect of RSV on Intracellular ROS

**Figure 3:**
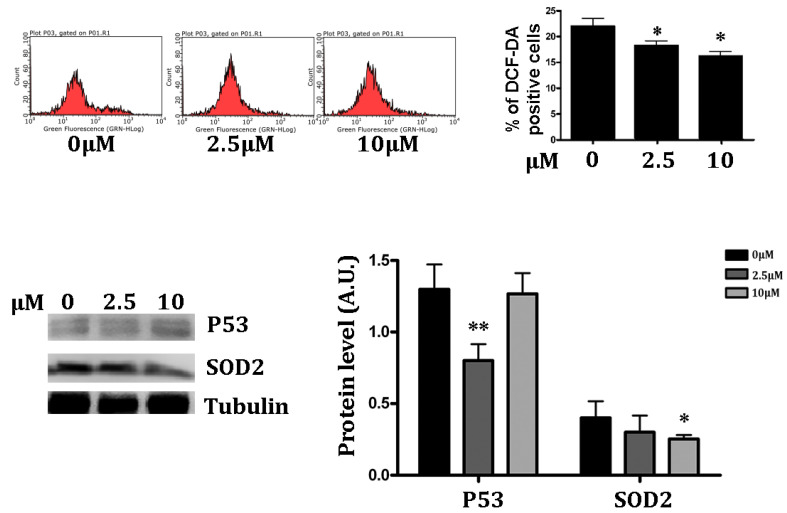
*RSV treatment increases intracellular antioxidant potential*. **Upper Panel**—RSV-treated and control PC12 cells were harvested after 24 h post hypoxia and then stained with DCF-DA fluorescent dye. The FACS analysis results are represented in % positive cells in the bar graph. Data were represented as means ± SD (*n* = 3), (* *p* <0.05, ** *p* <0.01). **Lower Panel**—The antioxidant potential of the cell was assessed by a protein expression analysis of SOD2 and p53 by Western blot. The blotted protein bands were quantified and represented in the bar chart, according to RSV treatment. Data were represented as means ± SD, (*n* = 3), (* *p* < 0.05, ** *p* < 0.001).

Hypoxia significantly increases the amount of ROS. Moreover, RSV promotes cytoplasm antioxidant capacity by increasing Superoxide Dismutase 2 (SOD2) and decreasing the lipid peroxidation [38]. We examined the antioxidant effect of RSV in PC12 cells in hypoxic conditions. Therefore, we used DCF-DA to test whether ROS formed in PC12 cells in hypoxic conditions, and whether RSV treatment altered hypoxia-induced ROS formation. We found that hypoxia treatment showed relatively high accumulation of free radicles (higher than 20%) in PC12 hypoxic cells, while incubation with 2.5 and 10 μM of RSV decreased the intracellular ROS contents in hypoxic PC12 cells (Figure 3, upper panel) (*p* < 0.05). The antioxidant activity of RSV is expressed in low doses: in fact, as shown in Figure 3, the lowest dose induces a significant decrease in ROS. In the Western blot analysis of p53 and SOD2, which are most influenced by elevated intracellular ROS, we found that the intracellular p53 expression was reduced at 2.5 μM (*p* < 0.01) in the RSV treatment group under hypoxic conditions. This observation may be correlated with the ROS-neutralizing potential of RSV; indeed, low ROS might reduce DNA damage, resulting in low p53 expression.

The antioxidant effect of RSV was alternatively assessed by quantifying the SOD2 concentration through a Western blot analysis. As shown in Figure 3 (bottom panel), the quantitative expression of SOD2 was found to be reduced in RSV-treated cells in hypoxic conditions (*p* < 0.05).

### 3.4. Measurement of Intracellular ROS Levels

**Figure 4:**
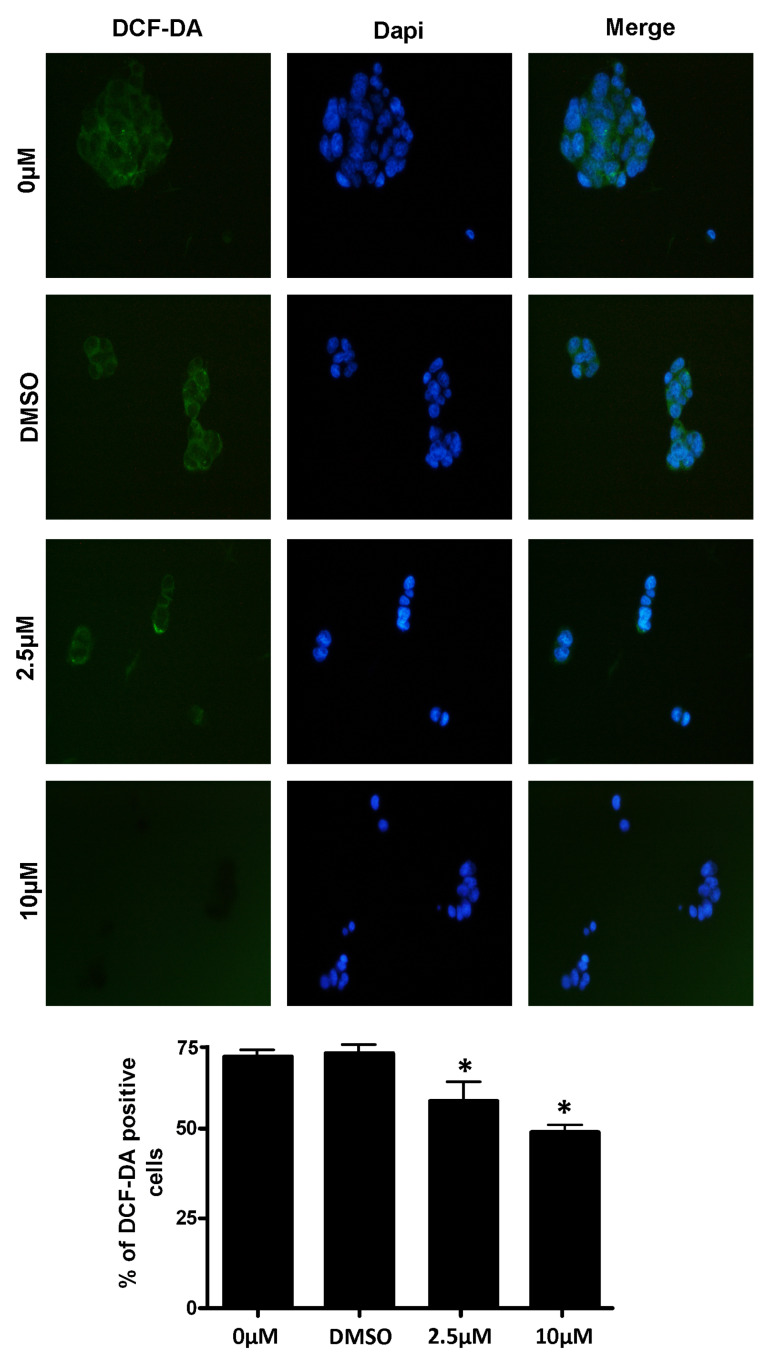
Measurement of intracellular ROS levels in RSV-treated PC12 cells under hypoxic conditions. Post-hypoxia and RSV treatment: the harvested PC12 cells were stained by CM-H2DCFDA to stain the intracellular ROS. DAPI was used to stain the nucleus. The images were obtained by a fluorescence microscope under low light conditions. The qualitative data of intracellular ROS in all concentration treatments of PC12 cells were captured through two different filters and a merged image was created. The lower panel contains the represented quantitative data, shown in the bar graph. Data were represented as means ± SD (* *p* < 0.05), of *n* = 3.

The intracellular generation of ROS was monitored in cells by the intensity of the green fluorescence staining of CM-H2DCFDA. The appearance of the RSV-treated control and hypoxia-exposed PC12 cells that were charged with the H2DCF fluorescent signal is reported in Figure 4, as observed by fluorescent microscopy. In the control cells (DMSO or 0 µM) the fluorescence signal was very high, and a clear intracellular fluorescence appearance was evident to show the increased accumulation of ROS. The intracellular fluorescent foci were observed to be weak, and they diminished in 2.5 µM and 10 µM RSV-treated stressed cells, respectively.

### 3.5. Cell Cycle Analysis

**Figure 5:**
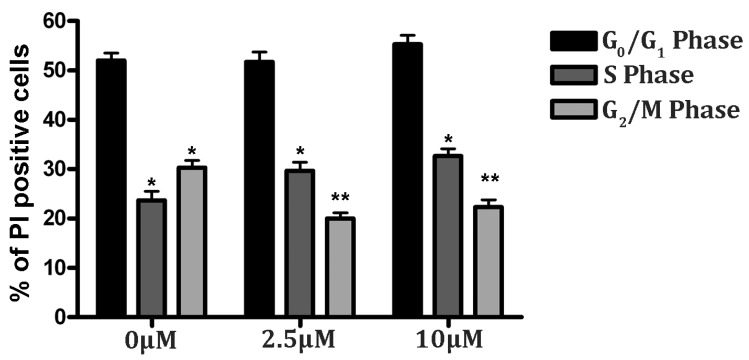
*RSV improves cell cycle progression in PC12 cells under hypoxic stress*. Cells were treated with RSV for 24 h prior to hypoxic treatment. Cells were cultured in fresh media and harvested post-24 h. Next, cells were fixed in ethanol and stained with PI for a cell cycle analysis by flow cytometry. Results of the cell cycle analysis were represented in percentage values (* *p* < 0.05, ** *p* < 0.01). The representative results of three independent experiments are shown in the bar graph. Data are represented as means ± SD of triplicate experiments.

A cell cycle analysis was performed to assess the progress of cellular development under oxidative stress conditions. The harvested cells were fixed in 90% chilled ethanol and stained with PI to analyze the DNA content and obtain the cell cycle stage through a FACS analysis. As shown in Figure 5, the RSV treatment in hypoxia did not induce significant cell changes in the G0/G1 phase. Interestingly, we observed an increased number of cells in the S phase, compared to the G2/M phase (higher than 30%) in the RSV-treated group under hypoxia. Precisely, the RSV groups (2.5 and 10 μM) showed 51.41% of cells in G1 and 29.29% in S; and 54.82% in G1 and 33.35% in S phase, respectively. Thus, although the cell population in the G0/G1 remains unchanged from the control (0 µM), the PC12 cells that are treated with RSV evidently resume the DNA synthesis process after 24 h of hypoxic stress, as shown in Figure 5 by the increase in the cell population in the S phase. This observation may be supported by the finding that RSV downregulates cell cycle regulator proteins, such as cdc2, cdk2, and cdk3, causing G1 arrest in four different cell lines [39].

## 4. Discussion

Cerebral ischemia is a common vascular disease worldwide, leading to deadly and disabling diseases such as cerebral infarction or hypoxic-ischemic encephalopathy without appropriate therapy. Brain stroke is the second leading cause of disability from the age of 50 [40] and, after coronary artery disease, constitutes the second most common cause of death worldwide [41]. Insufficient blood flow to tissue results in hypoxia, acute arterial thrombus formation and chronic narrowing of the arterial supply, which are the most critical factors contributing to the local or generalized deprivation of oxygen, which results in ischemia. For most neurodegenerative diseases there are no effective treatment options, due to ineffective passage across the blood–brain barrier and side effects [42]. The objective is to promote neuroprotective effects on the cellular signaling pathways at different stages of brain damage. The intervention time, pharmacokinetics, pharmacodynamics and activities of the compounds are critical to successfully counteract the consequences of stroke. The therapeutic window to reduce the pathological consequences of stroke, essentially neuronal damage, is estimated as 0–6 h for primary interventions [43,44] and may extend up to 24 h post-stroke. In recent decades, several studies have shown that RSV may act as a neuroprotective compound, but little is known about its potential in improving the cognitive and metabolic aspects that are associated with neurodegenerative diseases. RSV has been proved to be beneficial in a wide type of pathological conditions such as tumors, oxidative stress and myocardial ischemia-reperfusion injury [45]. In addition, resveratrol protected against spinal cord injury (SCI) via activating autophagy and inhibiting apoptosis through SIRT1/AMPK [46].

Hence, our study focuses on natural RSV with potent therapeutic properties and the ability to reach brain cells, in order to explore antioxidant and neuroprotective effects in a neuronal PC12 cell model. The PC12 cells are undifferentiated neuronal cells derived from rat pheochromocytoma and are the best suited in vitro experimental model for various neuronal developmental, as well as disorder, studies [47,48]. Some studies reported the induction of ischemic conditions in PC12 cells by using chemotoxicity to investigate the neuroprotective effect of RSV [47,48]. In our study, PC12 cells were subjected to OS, using a hypoxic chamber to mimic an ischemic condition. The neuroprotective effect of resveratrol and its underlying molecular mechanism was investigated solely under hypoxic conditions.

RSV is known to be a strong antioxidant with a high protective action against cytotoxicity and OS in neurons [49]. After evaluating the toxicity of RSV, two minimal concentrations (2.5 and 10 μM) of RSV were selected to check its antioxidant potential on the ischemic model, PC12 cells. We found that hypoxia reduced neuronal PC12 viability by inducing apoptosis and OS, while RSV treatment attenuated apoptosis and ROS-induced damage. Furthermore, the CM-H2DCFDA staining [50] of stressed PC12 cells, observed under the fluorescence microscope, showed the intracellularly absorbed RSV-induced dose-dependent inhibition of ROS, as observed by the decrease in green fluorescence of the ROS mediated oxidized CM-H2DCFDA dye.

RSV showed a dose-dependent attenuation of cell death under OS. Hypoxia induces a demand for redox potential in mitochondria; as a result, the leakage of free radicles from mitochondria initiates an intrinsic apoptotic pathway by expressing pro-apoptotic genes such as Bax, caspase-3, and caspase-9. Consistent with the reduction in apoptosis, we found a reduction in Bax and the activation of caspase-3 and caspase-9. Subsequently, we analyzed the ability of RSV to minimize the consequences due to the high concentrations of ROS that were generated in PC12 cells after hypoxic treatment, and we evaluated the expression profiles of key pro-apoptotic proteins.

The intrinsic apoptotic pathway is regulated by Bcl family proteins such as Bcl-2 and Bax, which play a key role in regulating the effect of mitochondrial membrane permeability, mitochondrial function and Cyt-c release [51,52,53]. Bcl-2 is mainly located in the nuclear, mitochondrial and endoplasmic reticulum membranes, while the proapoptotic Bax protein is mainly located in the cytoplasm. Bcl-2 can stabilize the barrier function of the mitochondrial membrane and inhibit the transfer of apoptosis-inducing factors to the nucleus [52,53]. Our study found that PC12 cells that were treated with RSV at 2.5 µM expressed low levels of Bax and Bcl-2 proteins, with a higher ratio compared to treatment with RSV at 10 µM. In contrast to the high Bax/Bcl-2 ratio, the activation of caspase-3 and -9 was greater with RSV at 10 µM. This result may indicate that apoptosis is not active, as confirmed by the flow cytometry analysis in which the percentage of apoptotic cells was less than the control. Consequently, Bcl-2 had a strong anti-apoptotic effect and the Bax-induced caspase-dependent apoptosis was inhibited, as demonstrated by a low level of caspase-3, -9 and, therefore, the survival of the PC12 cells. Similar observations were reported about the anti-apoptotic effect of RSV on SH-SY5Y cells [54], and other disease model systems [26,47].

Among human body cells, neurons are particularly vulnerable to an excessive free radical exposition, and OS is the major pathogenetic mechanism of neurodegenerative disorders. We analyzed the number of ROS-presenting cells by a DCF-DA-based fluorescent staining method. We observed that the treatment with resveratrol was able to significantly reduce the intracellular ROS production. This protective effect may be the reason for the reduced expression of SOD2, a key antioxidant enzyme. Furthermore, we found that RSV promoted growth, i.e., the progression of the cell cycle. The cell cycle analysis may indicate cellular health conditions after rescue from hypoxic stress. Interestingly, post-24 h of hypoxic treatment, the RSV-treated PC12 live cell populations were found in the G0/G1 and S phases of the cell cycle. The rising cell population in the S phase in the RSV group is indicates the rapid recovery of cellular health from oxidative stress.

## 5. Conclusions

In conclusion, these results suggest that RSV tends to implement the damage control pathways in neuronal cells under oxidative stress conditions. Hence, RSV has a protective effect in neuronal cells and may halt the cell cycle in the G1 or S phase to repair the intracellular damage. Therefore, RSV could be a good candidate to act as an antioxidant and a promising preventive therapeutic agent in neurodegenerative diseases for personalized medicine. More translational studies are needed to further analyze the molecular mechanisms of RSV, and in vivo studies are also needed to verify the antioxidant and antiapoptotic effects.

## Data Availability

Data are contained within the article.
